# Preliminary Study on EGCG-Enhanced Vanadium Toxicity in Cells: Impact on Oxidative Stress

**DOI:** 10.3390/molecules30102114

**Published:** 2025-05-09

**Authors:** Ewa Wnuk, Iwona Zwolak

**Affiliations:** Department of Biomedicine and Environmental Research, The John Paul II Catholic University of Lublin, Konstantynów Ave. 1J, 20-708 Lublin, Poland; iwona.zwolak@kul.pl

**Keywords:** EGCG, vanadium, oxidative stress, ROS

## Abstract

Environmental pollution by heavy metals (HMs) has become a serious threat in recent years due to their potential consequences for human health and life. One such metal is vanadium (V). Despite its numerous benefits—including antibacterial, antifungal, and anticancer properties—V induces cellular damage through oxidative stress. Epigallocatechin gallate (EGCG), a potent antioxidant found in large quantities in green tea, is considered an effective protector against the damaging effects of HMs on cells. The aim of this study was to evaluate the possible effect of EGCG on CHO-K1 cells exposed to V. This is the first experiment of its kind on healthy cells. Cells were treated with V and EGCG for 24 h, either in combination or separately. The doses were selected in a preliminary stage of the experiment (V 50 and 100 µM; EGCG 0.5 and 1 µM). As part of the study, the cell viability, total ROS activity, and mitochondrial membrane potential were assessed. The results showed that at the tested concentrations, EGCG did not reduce the toxic effect of V on cells, but in fact exacerbated its adverse effects on cells. Further studies are needed to understand the exact mechanism of V–EGCG interaction in mammalian cells.

## 1. Introduction

Industrial development, increased transport, and the excessive use of fertilizers in agriculture are just a few factors contributing to the increased levels of heavy metals (HMs) in the environment. Their presence has a number of consequences, including possible adverse effects on human health and life. The available literature shows that much of the research focuses on the presence of metals, such as lead (Pb), zinc (Zn), cadmium (Cd), and nickel (Ni), in the environment and their effects on humans. Given its properties and possible adverse effects, it is worth paying more attention to vanadium (V).

V is a transition metal. Its proximity to essential trace elements, as well as characteristics, such as rapid excretion, homeostatic regulation with accumulation, and its ability to catalyze the formation of biologically active compounds, make V, to some extent, an essential trace element [1]. It is required in small amounts for the proper functioning of the organism, making it one of the 49 essential nutrients [2]. In the environment, its most common forms are vanadyl (+IV) (VO_2_^+^), found in extracellular body fluids, and vanadate (+V) (VO_3_^−^), the intracellular form [3]. V (+V) is considered the most toxic, soluble, and stable form in the environment [4,5]. As documented, the toxicity of V compounds increases with the valence state [1,6]. The significant amounts of V in the environment come from volcanic emissions and marine aerosols. Additionally, the weathering of parent rock is thought to be the largest source of soil V contamination [1,7,8,9]. V exhibits antibacterial, antifungal, anticancer and anti-inflammatory properties [10]. A deficiency may lead to thyroid disfunction [11]. However, its toxicity requires further attention. In comparison with other HMs, this element is more toxic due to the high level of oxidative damage it causes to cells [6,12]. It is involved in the production of reactive oxygen species (ROS) during the bioreduction of vanadate to vanadyl, in mitochondrial interaction with V, and in vanadyl’s reaction with H_2_O_2_ [13,14]. Such reactions lead to oxidative damage induction, lipid peroxidation, and result in oxidative stress. An in vivo study showed that exposure to 5 mM vanadate affects the activity of antioxidant enzymes, including superoxide dismutase (SOD), glutathione peroxidase (GPx), and catalase (CAT) [15]. Moreover, V poisoning can adversely affect the heart, kidneys, gastrointestinal tract, and central nervous system [3,16].

The presence of heavy metals, including V, in the environment poses an ever-increasing threat to human health. There is an ongoing search for ways to protect the body from the harmful effects of these elements, such as by incorporating antioxidant-rich products into the diet. Green tea can be included in this group. This widely popular beverage, derived from *Camellia sinensis* and *C. assamica* plants, is known for its numerous medical benefits [17]. It exhibits antiapoptotic and anticarcinogenic properties and may act as an antioxidant and metal chelator [18,19,20]. These properties of green tea are attributed to its composition and, more specifically, to the presence of catechins, which belong to the flavonoid family and account for up to 25% to 35% of the green tea’s dry weight [17]. Among the eight major tea catechins, epigallocatechin gallate (EGCG) is the most abundant, making up to 70% of green tea catechins [21,22,23]. EGCG is believed to be responsible for most of green tea’s beneficial properties. Due to its antiproliferative properties and ability to induce apoptosis, EGCG is considered a potential therapeutic agent in cancer treatment. This has been confirmed by numerous in vitro studies on several types of cancer, including breast, lung, prostate, colorectal cancer, and melanoma. Moreover, many studies have reported its anti-infective, antiviral, anti-inflammatory, and antifungal properties. It also exhibits strong antioxidant and metal chelating properties, which have been proven in studies on animal and cell culture models [17,24,25]. However, despite its many known benefits, there are also reports showing completely opposite, pro-oxidative properties of EGCG [26,27].

The aim of this study was to evaluate the effect of EGCG, which is considered to be a powerful and effective antioxidant compound of natural origin, at doses achievable in the human body, on the pro-oxidant effect of V. Given the widespread occurrence of V and its possible negative effects on the body, it is reasonable to search for a compound that protects cells from the harmful effects of this metal. Due to the very limited number of reports on V–EGCG interactions under in vitro culture conditions, the research presented here is of a preliminary nature.

The research was conducted on Chinese hamster ovary (CHO-K1) cells, which are widely used in toxicological studies, such as for evaluating the cytotoxicity of organic and inorganic heavy metal compounds [28,29,30,31,32]. These cells are immortalized, non-cancerous cells characterized by a short doubling time and high proliferation efficiency [33,34,35]. Moreover, the CHO-K1 cell line is very often used in in vitro studies due to the ease of culture and maintenance and the absence of difficult culturing conditions and procedures. Due to the abovementioned features, the use of this cell line allows for obtaining repeatable research results [30,31].

## 2. Results

### 2.1. Effect of EGCG on V Toxicity in Cells

**Figure 1:**
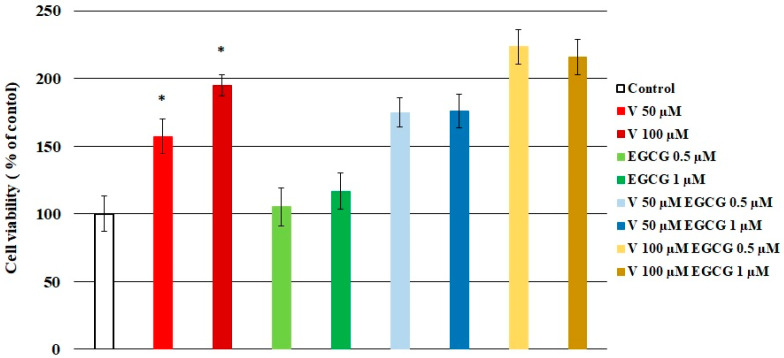
Effect of V, EGCG, and V + EGCG on CHO-K1 cell viability, measured with resazurin assay. The absorbance of resazurin in control cells was taken as 100%. Results are presented as a percentage of control cells and represented as mean + SD derived from three independent experiments. Cytotoxicity is indicated by an increase in percentage values, compared to the control cells; * significantly higher than the control (Mann–Whitney U test, *p* < 0.05).

The results of the study showed a decrease in cell viability, as indicated by an increase in absorbance values. Moreover, in cells treated with V (50, 100 µM) alone, a significant increase in cytotoxicity was observed (*p* < 0.05 M–W test), compared to the control samples. However, in the case of cells treated simultaneously with V and EGCG, no protective effect of EGCG on cells was observed (Figure 1). As observed, the simultaneous treatment of cells with V–EGCG caused a decrease in cell viability of about 20% in the case of V 50 µM and of about 30% in case of V 100 µM, in both tested EGCG concentrations.

### 2.2. Effect of V–EGCG Cotreatment on MMP

**Figure 2:**
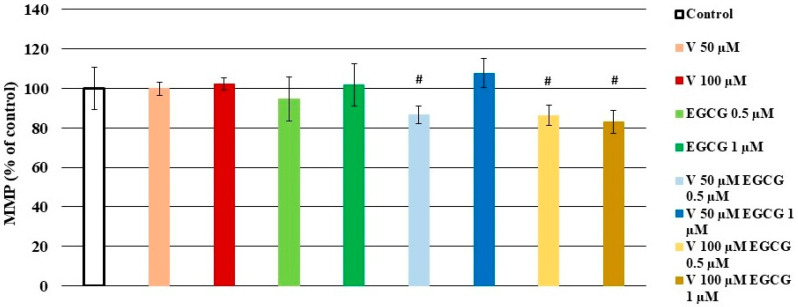
Effect of V, EGCG, and V + EGCG on mitochondrial membrane potential. The MMP value in control cells was taken as 100%. Results were expressed as a percentage of control cells—the higher the value, the higher the content of healthy cells. Results are represented as mean + SD derived from three independent experiments; # significantly higher than V alone (Mann–Whitney U test, *p* < 0.05).

Figure 2 presents the results for MMP in cells exposed to V, EGCG, and both reagents simultaneously after 24 h of incubation. After this period, a significant (*p* < 0.05 M–W test) decrease in MMP was observed in almost all variants treated simultaneously with V and EGCG (except for cells treated with V 50 µM- EGCG 1 µM), compared to cells treated with V alone. These results suggest the additive effect of EGCG at the applied concentrations. As observed in the case of V 100 µM samples, the increasing concentration of EGCG caused intensification of the toxic effect of V, manifested by a decrease in MMP values.

### 2.3. EGCG Influenced the ROS Production in CHO-K1 Cells Treated with V

**Figure 3:**
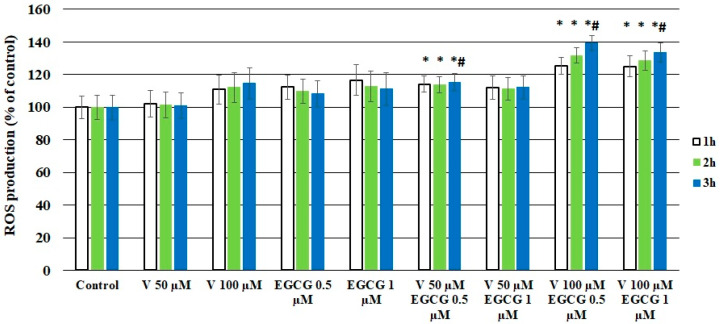
Effect of V, EGCG, and V + EGCG on ROS production after 1 h, 2 h, and 3 h of incubation. The fluorescence of the Total ROS Green dye in control cells was taken as 100%. Results were expressed as a percentage of control cells- the higher value, the higher the content of ROS in cells. Results are presented as mean + SD derived from three independent experiments; * significantly higher than the control; # significantly higher than V alone (Mann–Whitney U test, *p* < 0.05).

The results of the assay showed an increase in the fluorescence signal in most samples, after 1 h, 2 h, and 3 h of incubation. An exception was observed in cells treated with V 50 µM, where results comparable to those of the control samples were obtained. Moreover, coincubation with V 50 µM- EGCG 0.5 µM, V 100 µM- EGCG 0.5 µM, and V 100 µM-EGCG 1 µM caused a significant increase in ROS production compared to control cells. Furthermore, for results obtained after 3 h of incubation, a significant effect on ROS production was also observed in comparison with cells treated with V alone (*p* < 0.05 M–W test) (Figure 3).

### 2.4. Caspase-3 Activity

**Figure 4:**
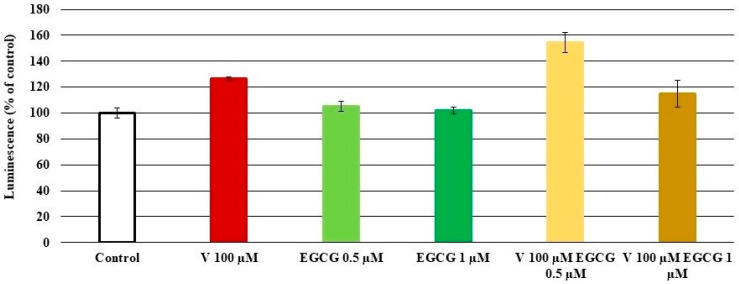
Effect of V, EGCG, and V + EGCG on caspase-3 activity. The luminescence of the control cells was taken as 100%. Results are presented as a percentage of control cells—the higher the value, the higher the caspase-3 activity. Results represented as mean + SD derived from three independent experiments. Lack of significance in the presented results.

The caspase-3 activity results in cells treated with V (100 µM) and EGCG (0.5 and 1 µM) are presented in Figure 4. A slight increase in activity was observed in cells treated with the tested reagents alone compared to control cells. However, in variants treated with V–EGCG together, no protective effect of EGCG was observed. The highest increase was observed in the V 100 µM-EGCG 0.5 µM variant. In the case of cells treated with V 100 µM- EGCG 1 µM, an insignificant slight decrease in luminescence was observed compared to cells treated with V 100 µM alone. The results did not show statistical significance (*p* < 0.05 M–W test).

All results have been collected and presented in the form of a table showing trends (increase/decrease) in the values obtained in individual tests for each variant. The results are presented in the Supplementary Material.

## 3. Discussion

This study presents the results of an investigation into the effect of EGCG on CHO-K1 cells treated with V. The dose of EGCG and V was chosen to approximate the natural concentrations found in the body [36,37,38,39,40]. This is the first in vitro study of V–EGCG interactions where both agents have been used at physiological doses. The results show that EGCG, at the doses tested, not only fails to attenuate, but actually enhances V toxicity. This was evidenced by decreased cell viability, increased ROS production, decreased MMP in almost all variants (except the V 50 µM-EGCG 1 µM sample), and activation of caspase-3. Moreover, the negative impact of EGCG-only treatment was also noted.

It has been repeatedly shown that the toxicity of V mainly results from the generation of oxidative stress. This has been demonstrated in both in vivo and in vitro studies. For example, it has been shown that sodium metavanadate stimulates the production of ROS and negatively affects GSH levels in the brains of rats [41]. In turn, in vitro studies conducted on the human lung epithelial cell line A549 observed the stimulation of hydroxyl radicals, H_2_O_2_, and superoxide anion production under the influence of metavanadate. Additionally, it was observed that ROS produced under the influence of vanadium caused cell cycle arrest in the G(2)/M phase [42]. In our analyses, an increase in ROS production was also observed in cells exposed to V. At a dose of 50 µM, a 5% increase in ROS production was observed compared to the control. A twofold increase in the concentration of V (100 µM) resulted in an increase of more than 10% in the amount of ROS produced by cells compared to control samples. Considering the numerous sources of vanadium in the environment and its negative impact on organisms, it is reasonable to search for compounds that can protect cells from its adverse effects. EGCG, due to its properties, seemed to meet these criteria.

It is difficult to find reports on the protective or toxic effects of EGCG on V-treated cells in the available literature. However, there are studies demonstrating the increased toxicity of other heavy metals under the influence of EGCG. For example, Nakazato et al. [43] observed that coincubation of EGCG and As in myeloma cells and Burkitt’s lymphoma cells resulted in reactions suggesting an increase in metal toxicity. Coincubation with 10 µM EGCG for 3 and 24 h resulted in decreased cell viability, elevated ROS production, and the activation of caspase-3, as observed in our study. Analogous results were also observed by Kim et al. [44], where EGCG (20 µM) promoted As-induced toxicity in primary bovine aortic endothelial cells (BAECs). Notably, the authors found that using these factors separately did not cause such an effect. The enhancement of the pro-oxidant effect by EGCG was also observed in the case of Cd. In one study, 10 µM Cd was shown to suppress the viability of PC-3 (human prostate cancer) cells, as measured by the MTT assay after 24 h of incubation, and the effect was significantly stronger when Cd and 50 µM EGCG were used together [45]. Additionally, it has been shown that EGCG and Cd directly affect mitochondria. One of the effects is the loss of mitochondrial membrane potential (MMP) [46]. The decrease in MMP was also noted in our experiment. A decrease in MMP was observed in cells treated with V 50 µM/EGCG 0.5 µM, V 100 µM/EGCG 0.5 µM, and V 100 µM/EGCG 1 µM of 13.5%, 13.7%, and 17.1%, respectively. In the case of the variant V 50 µM/EGCG 1 µM, an increase in MMP of nearly 8% was observed. Similar cell reactions to the action of a pro-apoptotic factor have already been observed in in vitro studies. Hyperpolarization was observed, for example, in Jurkat human leukemia T cells after camptothecin, H_2_O_2_,or Fas treatment [47,48] or after exposure to low-density lipoproteins in Caco-2 intestinal cells [49]. According to some authors, such a reaction is typical before common apoptotic events, such as caspase-3 activation or the breakdown of MMP [48]. Moreover, elevated MMP is a protective mechanism of the cell which may or may not result in apoptosis [50]. Furthermore, enhanced Cd toxicity in the presence of EGCG was noted in cells derived from transplantable rat pheochromocytoma (PC12 cells) [51]. The authors showed that 1.5 µM EGCG combined with 5 µM Cd resulted in decreased cell viability (measured using the trypan blue test), which was associated with cell membrane damage. As explained by several authors, the reduction in cell viability influenced by heavy metal + EGCG interaction may result from cell growth inhibition affected by catechin–metal ion interactions which lead to an imbalance in cell metabolism, or increased heavy metal content in cells caused by the direct reaction of these two compounds with each other.

On the basis of the available studies, it can be concluded that EGCG is characterized by antioxidative, anti-inflammatory, chemopreventive, and anticancer properties. However, there are reports indicating that EGCG may also have completely opposite effects, negatively affecting both cancerous and normal cells. For instance, it was shown that EGCG treatment for 24 h at a concentration of 100 µM caused a 25% decrease in the viability of human peripheral lymphocytes [52]. Additionally, Nakagawa et al. [53] observed a 50% inhibition of murine osteoclast cell growth when 58 µM EGCG was used for 24 h. A similar degree of growth inhibition was observed in human bone marrow mononuclear cells treated with 100 µM EGCG for 14 days [54]. Such negative effects of EGCG were also noted in our study. Specifically, cell supplementation with 0.5 and 1 µM EGCG resulted in a 5% and 17% decrease in cell viability, respectively, compared to untreated cells. Moreover, increased ROS production was also observed. In the case of MMP, a negative effect was observed at the lower EGCG dose, which caused only a slight decrease in MMP, indicating cell damage. EGCG cytotoxicity was also observed in cancer cells. For example, the MTT assay showed a 50% decrease in the viability of HT-29 cells after treatment with EGCG (up to 200 µM for 48 h) [55]. Analogous susceptibility to EGCG was also observed in human peripheral lymphocytes and the Nalm-6 cell line at concentrations of 10–60 µM [42], as well as in KYSE510 (human esophageal squamous cell carcinoma) cells treated with 20 µM EGCG [56].

## 4. Materials and Methods

### 4.1. Reagents

Fetal bovine serum (FBS, cat. No. F9665), antibiotic antimycotic solution (100×) (cat. No. A5955), trypsin–EDTA solution (0.05%) (cat. No. T3924), sodium metavanadate (NaVO_3_, cat. No. 590088), (−)-epigallocatechin gallate (EGCG, cat. No. E4143), and an In Vitro Toxicology Assay Kit, Resazurin-Based (cat. No. TOX8-1KT) were purchased from Sigma-Aldrich (St. Louis, MO, USA). Dulbecco’s modified Eagle’s medium/nutrient mixture F-12 (DMEM/F-12) (cat. No. 31330095) was purchased from ThermoFisher Scientific (Waltham, MA, USA). The Caspase-Glo 3/7 Assay Kit (cat. No. G8090) was purchased from Whitehead Scientific, Promega (Madison, WI, USA). The Intracellular Total ROS Activity Assay Kit (cat. No. 9144), Hydrogen Peroxide Fluorescent Detection Kit (cat. No. 9131), and MitoPT^®^ TMRM Mitochondrial Depolarization Assay Kit (cat. No. 9105) were purchased from ImmunoChemistry Technologies (Bloomington, MN, USA).

### 4.2. Cell Culture

The CHO-K1 cell line (cat. No. 85051005) was purchased from Sigma-Aldrich (St. Louis, MO, USA). Cells were cultured in a humidified incubator (CO_2_ incubator HERAcell 150i, Thermo Scientific, Rheinfelden, Germany) at 37 °C and 5% CO_2_, in DMEM/F-12 containing 5% FBS, 100 U/mL penicillin, 100 mg/mL streptomycin, and 0.25 µg/mL amphotericin B. Cultures were passaged three times a week using a 0.05% trypsin solution. The cells were monitored using a phase-contrast microscope (Olympus, model IX73, Tokyo, Japan). All procedures requiring sterile conditions were carried out in a laminar flow cabinet (Herasafe laminar flow cabinet, model KS, Thermo Scientific, Germany).

### 4.3. V and EGCG Concentrations

In this study, V and EGCG were used at two concentrations, namely 50 and 100 µM for NaVO_3_ and 0.5 and 1 µM for EGCG. Concentrations of V and EGCG were selected based on microscopic analysis of the cells under the influence of different concentrations of the agents (Supplementary Figure S1). Higher concentrations than those selected led to a significant decrease in cell viability or necrosis of the majority of cells in the population after 24 h of incubation.

### 4.4. Reagents Preparations

The EGCG was dissolved in 0.1% DMSO to obtain a final concentration of a 20 µM stock solution. It was then partitioned into smaller volumes and frozen. The stock solution was used to prepare the tested concentrations of EGCG just before addition to the culture media.

The NaVO_3_ stock solution was prepared in deionized water at a concentration of 10 mM and kept at 4 °C. The solution was colorless and was used before cell induction.

### 4.5. Cell Viability Assessment with Resazurin

To evaluate the cytotoxic effect of V, EGCG, and their interaction, the In Vitro Toxicology Assay Kit, Resazurin-Based (cat. No. TOX8-1KT; Sigma-Aldrich) was used. Resazurin is a non-toxic redox dye that remains stable in culture medium. In this assay, blue and nonfluorescent resazurin is reduced to pink and highly fluorescent resorufin by mitochondrial oxidoreductases, which are produced by living cells [32]. The intensity of the colour change to pink is proportional to the number of living cells.

CHO-K1 cells were seeded at 1 × 10^4^ cells/well into 96-well plates containing DMEM/F-12 with 5% FBS and cultured for 24 h at 37 °C in 5% CO_2_. After 24 h, the old medium was replaced with fresh medium containing EGCG (0.5 and 1 µM) and NaVO_3_ (50 and 100 µM), either in combination or alone. After another 24 h, the medium was replaced with fresh DMEM/F-12 without FBS, and 10 µL of resazurin was added. The cells were then incubated for 3 h. Absorbance was measured using a microplate reader (Synergy 2, BioTek Instruments Inc., Winooski, VT, USA) at 600 nm (with a 690 nm background correction). Data from the resazurin assay are expressed as a percentage of control cells. They were calculated as follows:

(A_test_/A_control_) × 100%

where A_test_ represents the absorbance of cells treated with V or EGCG, and A_control_ represents the absorbance of control cells. Cytotoxicity (cell injury) was indicated by an increase in percentages compared to control cells.

### 4.6. Mitochondrial Membrane Potential (MMP) Assessment with MitoPT TMRM Assay

For mitochondrial membrane potential (MMP) measurements, the MitoPT TMRM Assay Kit (cat. No. 9105; ImmunoChemistry Technologies, Davis, CA, USA) was used, with TMRM (tetramethylrhodamine methyl ester) as the fluorescent dye. In this assay, the fluorescent dye accumulates in healthy cells with intact membrane potential and emits red–orange fluorescence. In damaged cells, mitochondria lose their membrane potential, leading to reduced TMRM accumulation and a decay in fluorescence emission. As a result, healthy cells generate higher fluorescence unit outputs of orange fluorescence compared to apoptotic ones.

For the assay, cells were seeded at 1 × 10^4^ cells/well into 96-well plates containing DMEM/F-12 with 5% FBS and cultured for 24 h at 37 °C in 5% CO_2_. After 24 h, the cells were treated with V, either alone or in combination with EGCG, at the same concentrations as previously described, for 24 h at 37 °C in 5% CO_2_. MMP was then determined according to the manufacturers’ instructions. Accordingly, the supernatant was replaced with 200 nM MitoPT working solution, and the cells were incubated for 30 min at 37 °C in the dark. The cells were then washed with assay buffer and placed in a fresh portion of assay buffer until analysis. Fluorescence intensity was measured using a microplate reader (Synergy 2, BioTek Instruments, Inc., USA) after 1 h and 24 h, with excitation and emission wavelengths of 530 nm and 590 nm, respectively.

### 4.7. Determination of Changes in ROS Levels Using an Intracellular Total ROS Activity Assay Kit

To evaluate the amount of ROS produced by cells treated with V and EGCG, separately and in combination, the Intracellular Total ROS Activity Assay Kit (cat. No. 9144; ImmunoChemistry Technologies) was used. In this assay, the Total ROS Green reagent is oxidized from a non-fluorescent to a fluorescent molecule in the presence of ROS produced by cells.

For the assay, cells were seeded at 1 × 10^4^ cells/well into 96-well plates containing DMEM/F-12 with 5% FBS and cultured for 24 h at 37 °C in 5% CO_2_. After 24 h, the medium was removed, and cells were incubated for 30 min in ROS Green in a 1:50 *v*/*v* ratio. Subsequently, the cells were washed with pure DMEM/F-12 and then treated with V (50 and 100 µM), either alone or in combination with EGCG (0.5 and 1 µM), for 1 h at 37 °C in 5% CO_2_ in the dark. The fluorescence signal was measured using a microplate reader (Synergy 2, BioTek Instruments, Inc., USA) using excitation and emission wavelengths of 485 and 528 nm, respectively. Measurements were taken after 1 h, 2 h, and 3 h of incubation with the fluorescence dye. Data (ROS levels) were expressed as a percentage of the control cells.

### 4.8. Determination of Caspase-3 Activity

Caspase-3 activity was determined using the Caspase-Glo 3/7 Assay Kit (cat. No. G8090; Whitehead Scientific, Promega). The assay involves generating a luminescent signal by luciferase from the provided luminogenic reagent. The activity of caspase increases as the apoptosis process progresses, and the resulting signal is proportional to the caspase activity.

For the assay, cells were seeded at 1 × 10^4^ cells/well into 96-well plates containing DMEM/F-12 with 5% FBS and cultured for 24 h at 37 °C in 5% CO_2_. After 24 h, the cells were treated with V and EGCG, either alone or in combination, using the highest V concentration (100 µM), for 3 h at 37 °C in 5% CO_2_ in the dark. Subsequently, the Caspase-Glo 3/7 Assay was performed according to the manufacturer’s protocol. Luminescence signals were measured after 30 min of incubation using a microplate reader (Synergy 2, BioTek Instruments Inc.).

### 4.9. Statistical Analysis

The results presented in this paper were obtained from three independent experiments, in which each analyzed variant was performed in six repetitions. This made it possible to verify the reliability of the results obtained

Data were analyzed using the Statistical Package for the Social Sciences (IBM Corp. Released 2020. IBM SPSS Statistics for Windows, Version 27.0. IBM Corp, Armonk, NY, USA). First, all datasets were checked for outliers using the Tukey fence method. A Mann–Whitney U (M–W) test was then performed to detect the effects of V, EGCG, and the combination of V and EGCG on the measured parameters.

## 5. Conclusions

The presented results show that EGCG, at the doses used in this study, did not have a protective effect on V-treated CHO-K1 cells. Moreover, the analyses carried out showed an enhancement of the toxic effect of this element. This was evidenced by a decrease in cell viability, a reduction in MMP, and an increase in ROS production in cells treated with both agents simultaneously. Additionally, a negative effect on cells treated with EGCG alone was also observed. Given the limited number of reports on this topic, further analysis is necessary to understand the exact mechanism underlying the EGCG–V interaction and its effect on cells.

## Data Availability

Data is contained within the article and Supplementary Materials.

## Supplementary Materials

The following supporting information can be downloaded at: https://www.mdpi.com/article/10.3390/molecules30102114/s1, Figure S1: The first step was the observation of cells under the phase-contrast microscope. After 24 h the control samples (a) were characterized with dense monolayer of cuboidal/polygonal cells. The 24 h with V tested concentrations-50 µM (b), 100 µM (c)-showed a decrease in the cells density and, starting from the 150 µM concentration (d), changes in shape-cells were rounded. In the case of EGCG-0.5 µM (e), 1 µM (f), 5 µM (g)-such reaction was observed at the 5 µM concentration; Table S1: The table presents the general trends in the results obtained from tests on MMP, ROS production, and the viability of CHO-K1 cells subjected to V (50 and 100 µM), EGCG (0.5 and 1 µM), separately and together. The blue color indicates a statistically significant lower result, while the red color indicates a statistically significant higher result compared to the control (*p* < 0.05, M-W test).
