# Supplementary material for: Preliminary Study on EGCG-Enhanced Vanadium Toxicity in Cells: Impact on Oxidative Stress
Source: Molecules. 2025 May 9;30(10):2114. doi: 10.3390/molecules30102114 (PMC12113699; doi:10.3390/molecules30102114)
Supplement: Supplementary file 1 [file molecules-30-02114-s001.zip › Supplementary material/Supplementary Material Table1.pdf]

Supplementary Table 1. The table presents the general trends in the results obtained from tests on MMP, ROS production, and the viability of CHO-K1 cells subjected to V (50 and 100 µM) , EGCG (0.5 and 1 µM), separately and together. The blue color indicates a statistically significant lower result, while the red color indicates a statistically significant higher result compared to the control ($p < 0.05$, M-W test).

	V 50µM	V 100 µM	E 0.5 µM	E 1 µM	V 50 µM<br>E 0.5 µM	V 50 µM<br>E 1 µM	V 100 µM<br>E 0.5 µM	V 100 µM<br>E 1 µM
Viability	↑	↑	↑	↑	↑	↑	↑	↑
MMP	↑	↑	↑	↑	↑	↑	↑	↑
ROS	↓	↑	↓	↑	↓	↑	↓	↓
