# Supplementary figures and images for: Preliminary Study on EGCG-Enhanced Vanadium Toxicity in Cells: Impact on Oxidative Stress
Source: Molecules. 2025 May 9;30(10):2114. doi: 10.3390/molecules30102114 (PMC12113699; doi:10.3390/molecules30102114)

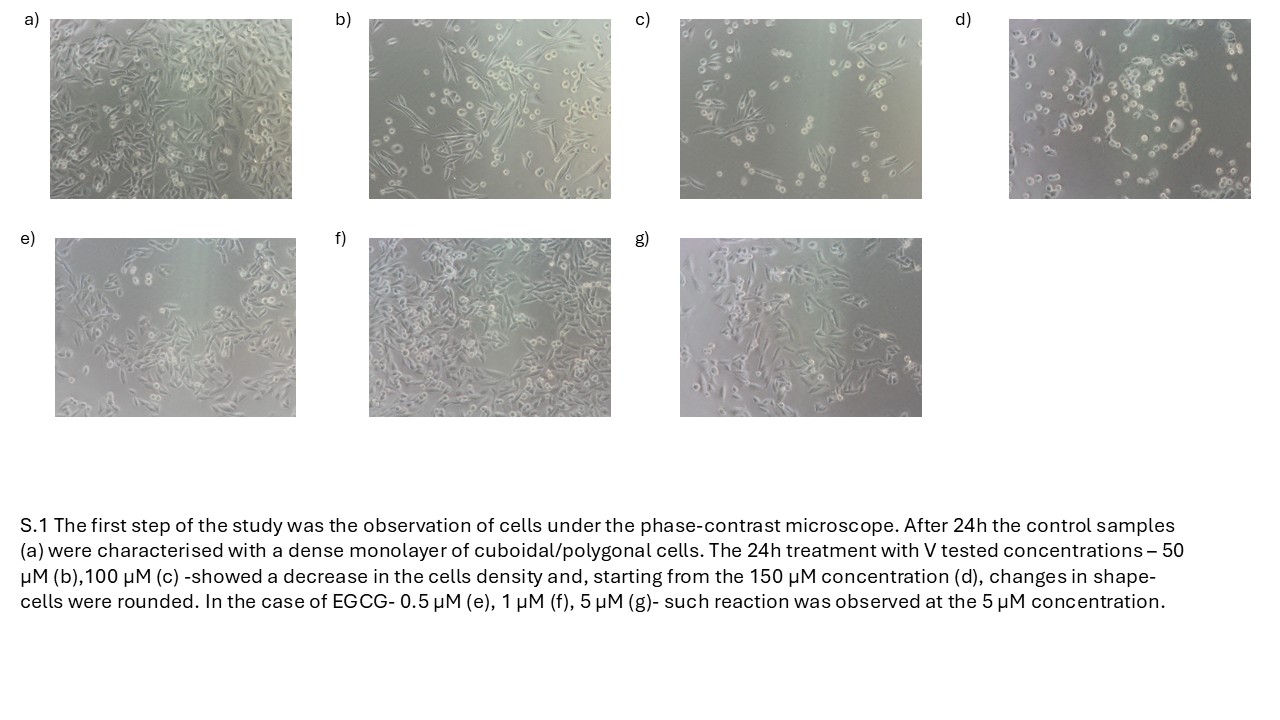

Supplement: Supplementary file 1 [file molecules-30-02114-s001.zip › Supplementary material/Supplementary Material Figure S1.jpg]
